# Quantifiable Cross-cultural Research on Medical Mistrust is Necessary for Effective and Equitable Vaccination in Low- and Middle-income Countries

**DOI:** 10.1007/s44197-024-00319-0

**Published:** 2024-10-28

**Authors:** Sean P. Prall

**Affiliations:** Department of Anthropology, University of California, Los Angeles, Los Angeles, CA USA

**Keywords:** Medical Mistrust, Vaccination, Health Inequalities

## Abstract

Perceptions of healthcare personnel and institutions substantially impact healthcare behaviors. In the US, minority experiences with racist events like the Tuskegee study, alongside everyday experiences of marginalization and discrimination, drive medical mistrust in populations that are already burdened with health inequalities. However, the concept of medical mistrust is rarely applied outside of industrialized contexts. Histories of colonialism, underfunded healthcare institutions, and the enormous cultural and ethnolinguistic diversity present in low- and middle-income countries (LMICs) make medical mistrust a likely contributor to health behavior in these contexts. In the era of COVID-19 and emergent malaria vaccines, there is an urgent need to mitigate factors leading to medical mistrust, which impedes interest in novel vaccines. Doing so requires substantial investment in research that examines the causes of medical mistrust across diverse communities, develops methodological tools that can effectively measure medical mistrust across diverse cultural and ethno-linguistic contexts, and applies this data to policy and public health messaging. This commentary highlights the role of medical mistrust in vaccination and argues for its utility in addressing vaccine decision-making in LMICs.

## Introduction

Vaccines represent one of the greatest technological innovations for addressing infectious diseases and preventing millions of deaths each year. Following the development of COVID-19 vaccines, multiple malaria vaccines, RSV, Ebola, dengue, and the ongoing development of vaccines for HIV, various cancers and non-infectious diseases, some have argued that we are currently in the “golden age of vaccines” [1]. Simultaneously, following the COVID-19 pandemic, we are seeing a resurgence and expansion of anti-vaccine rhetoric [2, 3]. According to UNICEF, while the response to the COVID-19 pandemic was characterized by many technological and public health successes, it also spurred mistrust and misinformation that spread beyond COVID-19 and led to a decrease in public support for childhood vaccines [4]. As we continue to make technological progress in fighting infectious diseases, we must also consider that these developments are only successful when people elect to participate. The swift resurgence in anti-vaccine sentiment highlights broader problems of mistrust in healthcare personnel and institutions. Considering sociocultural influences on decision-making is particularly important for countering health disparities in underserved contexts where vaccine uptake is widely variable, and distrust of healthcare systems is often endemic.

Reasons for vaccine hesitancy are numerous and well described [5]. However, some have criticized the public health discourse surrounding vaccine decision-making as an overly simplistic dichotomy between receiving adequate health information and exposure to anti-vax conspiracy information [6–9]. Instead, Leach et al. [7] argue that vaccine hesitancy should be relabeled as vaccine anxiety. This reframing allows for a wider conception of the cultural drivers of vaccine decision-making, not solely driven by access to the correct type of vaccine information. Medical mistrust, a concept largely developed and applied in the USA, directly assesses social and political influences on healthcare decision-making. Defined as “a tendency to distrust medical systems and personnel believed to represent the dominant culture in a given society” [10], this concept incorporates group and cultural-level interactions and assumes that these interactions directly impact health decisions. In addition to vaccination, medical mistrust has been applied to perceptions of healthcare personnel, healthcare utilization, routine screenings, follow-up care, and participation in clinical trials. Unlike dominant concepts that rely on information, knowledge, and access, such as the WHO’s recent focus on “infodemic management” [11], medical mistrust reflects larger social issues that may lead to a tendency to believe and act on information or misinformation, altering healthcare decisions and potentially exacerbating health inequalities.

Medical mistrust as a driver for healthcare decisions is typically used to understand African American health inequalities, and with few exceptions has rarely been applied outside of the US. However, this concept has great potential to explain healthcare behaviors, including vaccination, from a global health perspective. Incorporating the concept of medical mistrust into vaccine behavior studies may be particularly fruitful in low- and middle-income countries (LMICs). In particular, countries with particularly high ethnolinguistic diversity, historical experiences with colonialism and domination, and with minority groups interacting with a majority-outgroup may benefit from more nuanced understandings of how beliefs about and interactions with healthcare services shape vaccine decision-making. Additional research that better integrates cultural diversity and measures the experiences and impacts of medical mistrust can help address health disparities in low- and middle-income countries. In the “golden age” of vaccines, this work is increasingly urgent and necessary.

## Medical Mistrust Impacts Health Behavior and Vaccine Decisions

A sizeable body of evidence has shown that medical mistrust negatively impacts health behavior. This includes feelings, beliefs, and decisions about healthcare and healthcare personnel. Much of the early work was focused on understanding African American experiences with healthcare systems, as researchers were keen to understand how the fraught history of racism and discrimination in the American healthcare system, including notorious events like the Tuskegee Syphilis Study, impacted healthcare decisions [12]. This work suggests that for many Black patients, mistrust is central to decision making. For example, LaVeist et al. [13] found that participants who mistrusted their medical system were more dissatisfied with care, and this association drove the relationship between race and perceptions of care. Since then, numerous studies have demonstrated links between medical mistrust and healthcare perceptions, including perceptions of quality, satisfaction with care, confidence in genetic testing, comfort, confidence, and satisfaction [10, 14, 15]. More recent research has expanded to include other minority populations, patients of mixed race, and sexual minorities in the US [10, 14].

In addition to perceptions of healthcare and healthcare providers, medical mistrust also mediates actual healthcare decisions. African American men with high medical mistrust are more likely to delay routine healthcare check-ups, cholesterol, and blood pressure screenings [16]. Delays in routine care can have negative downstream effects on health outcomes, and contribute to health inequalities between White and Black patients. Other work indicates that medical mistrust contributes to ignoring medical advice, postponing care, delaying testing and screenings, and participation in clinical trials [10, 14, 15, 17]. Medical mistrust has also been used to understand vaccination decisions and is associated with vaccine hesitancy and belief in vaccine misinformation [18–20]. In a sample of US respondents, Allen et al. [21] found that the odds of COVID-19 vaccination decreased by 16% for each additional point on the medical mistrust index, with similar findings replicated elsewhere. These studies make it clear that medical mistrust is at the root of racial disparities in health behaviors, perceptions of healthcare, vaccine hesitancy and misinformation, and vaccine decisions.

## Medical Mistrust in Low- and Middle-income Countries

To date, with few exceptions, the entirety of research on the impacts of medical mistrust takes place in the Global North, primarily in the US. In one recent review, only 1% of quantitative studies relied on a non-US sample [10]. There are notable additions to the literature; quantitative and qualitative studies that examine medical mistrust within communities and its impact on health [9, 19, 22–25]. However, these additions remain rare and are often culturally and methodologically idiosyncratic. In many LMICs, histories of colonialism, cultural diversity, and poverty make medical mistrust a particularly salient concept for understanding health behaviors.

LMICs have some of the highest levels of maternal and child mortality, the bulk of the world’s deaths from HIV and malaria, and substantial burdens from diarrheal and respiratory diseases. Given these considerable health needs, some scholars have puzzled over the low levels of preventative health behavior (e.g. bed nets, water treatment) [26]. However, this framing is problematic, implying that healthcare decisions and behaviors are the result of irrationality, ignoring socio-cultural and historical factors. Instead, there is evidence that healthcare decision-making has been shaped by the legacy of colonial interventions. Colonial medical campaigns in Africa were often brutal affairs, forcing Africans to undergo poorly executed and often dangerous medical testing and treatment. For example, French military teams forced villagers to undergo testing and treatment for African sleeping sickness using toxic drugs that caused blindness and actually increased the rate of death for those infected [27]. Sometimes participation in these campaigns was coerced at gunpoint. Lowes and Montero [28] examined the cultural-historical impact of these campaigns on modern-day health behaviors. They showed that historical exposure to the French sleeping sickness campaigns was associated with reduced vaccination rates and willingness to consent to a blood test (a proxy for trust in the medical provider). Further, they found that modern-day health campaigns in areas with a previous history of French colonial sleeping sickness campaigns were less successful, although this was not true of other, non-health-related projects. Similarly, Athias and Macina [29] found that historical exposure to the slave trade resulted in lower levels of childhood measles vaccination, which they link to familial transmission of mistrust. These findings point to a deep cultural history of mistrust of healthcare in sub-Saharan Africa, stemming from the legacy of colonialism that continues to impact Africans today.

Low- and middle-income countries, particularly those in Africa, also represent enormous cultural and ethno-linguistic diversity. However, economic analyses indicate that ethnic diversity is a poor predictor of health outcomes, which are better explained by indices of infrastructure development and public corruption [30]. Nevertheless, ethnic diversity may prime healthcare systems for medical mistrust, particularly when one ethnic group has been economically or politically dominant, and represents a disproportionately higher share of healthcare workers. For example, our work in Namibia indicates that Himba pastoralists report having poor experiences in hospitals partially because all healthcare workers are from other ethnic groups and are reported to discriminate against Himba [22]. These disparities can be exacerbated by linguistic barriers and gaps in education and health literacy [31]. More broadly, market integration, with increased access to formal education and healthcare can accentuate disparities between groups at the local level, resulting in distrust of doctors and nurses that represent the majority outgroup.

Medical mistrust doesn’t just undermine everyday healthcare behaviors and vaccination rates in Africa; it can have major impacts during infectious disease outbreaks. Perhaps the most infamous are the events leading to a boycott of polio vaccines in Northern Nigeria. Distrust of the polio vaccine was fomented through misinformation and historical suspicion of rival political groups, but also Pfizer’s 1996 “Trovan trial” and subsequent political fall-out, leaving Nigerians distrustful of vaccination [32]. As a result, Nigeria experienced a polio outbreak that spread to other countries, setting back regional efforts to eradicate the disease. Elsewhere, poor communication and engagement with communities during polio vaccination campaigns can lead to vaccination refusals and increase mistrust. In particular, incongruity between regular healthcare needs and access and door-to-door campaigns, poor information delivery about the purpose of vaccine campaigns, the frequency of campaigns leading to community fatigue, and lack of clarity of polio virus subtypes targeted by different vaccine efforts all undermine trust in vaccines, healthcare workers, and vaccination programs [33–35]. Similarly, institutional mistrust has been linked to failed mitigation efforts during Ebola outbreaks. A survey conducted during the 2018 Ebola outbreak in the Democratic Republic of Congo found that respondents with low institutional trust were less likely to adopt preventative health behaviors, accept Ebola vaccines, and seek healthcare treatment [36].

## The Problem with the Current State of the Literature

Applying medical mistrust to understanding disparities in health behaviors is clearly relevant in a global health context. However, significant problems exist with the current instruments. There are several surveys that have been widely used in the US, but they vary in the types of mistrust they measure [37]. The most commonly used are the group-based medical mistrust scale (GBMM), which focuses on ethnic group-level mistrust of medical personnel, and the medical mistrust index (MMI), which focuses on individual-level mistrust of health organizations and systems [17, 38]. Both were developed for US populations. In addition, nearly 20% of studies forego these validated indices for a single or small number of questions [37]. In a major review of medical mistrust studies, Benkert et al. [10] declare the literature “ubiquitous yet unclear,” with little consensus on which instrument is most appropriate, the analytical or conceptual framework whereby medical mistrust impacts health outcomes or even the nature of mistrust under study.

**Fig. 1:**
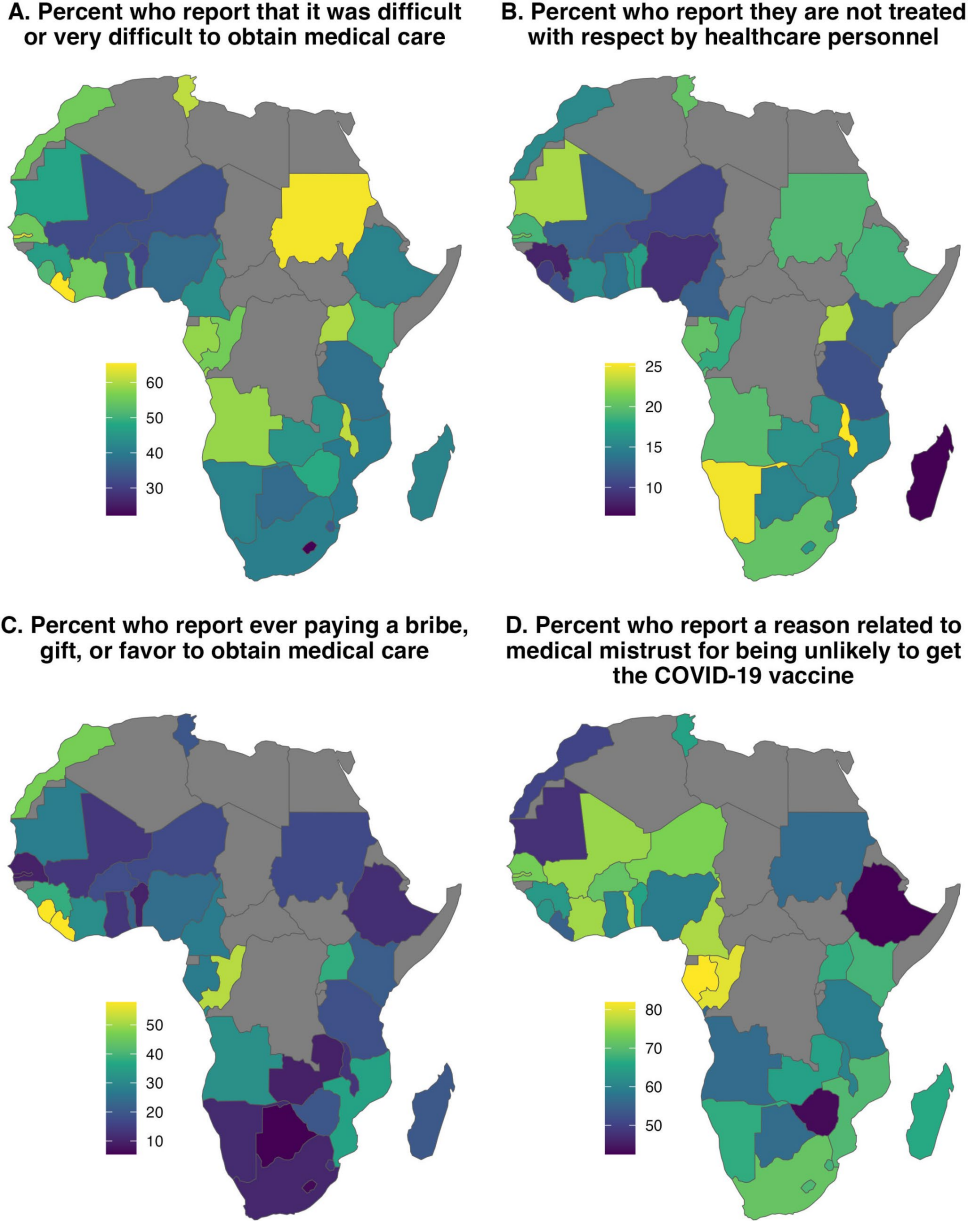
Afrobarometer round 9 data conducted in 39 countries (*N* > 53k, 2023) on items related to medical mistrust [39]. (**A**) Percentage of respondents by country who report that it was difficult or very difficult to obtain medical care or medical services. (**B**) Percentage of respondents by country who, when asked whether they feel they are treated with respect by healthcare personnel respond with the option “not at all.” (**C**) Percentage of respondents by country who report ever having to pay a bribe, gift, or do a favor to healthcare personnel in order to receive medical care or services. (**D**) Percentage of unvaccinated respondents who are uninterested in receiving the vaccine, and give an explanation of their lack of interest related to medical mistrust (e.g. COVID-19 doesn’t exist, lack of trust in vaccine, lack of trust in government, belief that the vaccine can cause disease, belief that the vaccine is part of experimentation, etc.). Items summarized in panel **A**, **B**, and **C** were only collected for participants who report that they had contact with a clinic or hospital in the past year. Item summarized in panel **D** was only asked of participants who reported not having received a COVID-19 vaccine and reporting being unlikely to try and get the vaccine if it was available (*N* = 12743). See afrobarometer.org for data, coding, and additional details on this survey

There are special considerations that need to be taken into account when developing and deploying a medical mistrust survey for LMICs. Current instruments such as the GBMM and MMI were not developed with ethnolinguistic diversity in mind, and as such are not necessarily cross-culturally valid. These instruments consist of numerous difficult-to-translate Likert scale items which may be challenging to re-interpret and translate in contexts where literacy rates are low. Many of the items in these surveys obliquely reference specific US events like Tuskeegee for engendering mistrust for a specific minority group (e.g. “Doctors and healthcare workers treat people of my ethnic group like ‘guinea pigs’”, an item from the GBMM survey). While areas in Africa may have similar notable effects, for many populations who have not experienced unethical medical testing these items are not relevant. Instead, other domains, such as corruption, bribery, and treatment by healthcare personnel may be more important drivers of mistrust (Fig. 1). In addition, there may be other important subdomains of medical mistrust not covered by the current instruments, but which are applicable for populations in diverse low-income countries. Qualitative research is a critical pathway toward understanding both known and unknown drivers of mistrust. In addition, qualitative studies can be used in conjunction with quantitative work, both to help design survey instruments and to interpret findings.

There is also general confusion between medical mistrust, generalized mistrust, “cultural mistrust” and “conspiracy beliefs” related to health-related information like infectious diseases and vaccination. Conspiracy beliefs about diseases and their origins are common and pernicious and may covary with medical mistrust. However, the assumption by some that conspiracy beliefs should be described as cultural barriers to healthcare because a particular ethnic group is more likely to espouse them is highly problematic and ignores the cultural-historical window through which information is interpreted [15]. Instead, suspicion of a majority outgroup with a deep history of maltreatment and discrimination is a logical protective response that should be corrected with non-biased healthcare systems, not minimized with dismissive labels [40]. A deeper analysis of the roots of conspiracy belief instead suggests that epistemic mistrust, instilled through historical violations of trust, racism, and trauma, drives psychological biases that lead to belief in misinformation [41]. Other evidence indicates that medical mistrust is indeed a singular concept, distinct from other types of mistrust [10]. Clarifying the causal pathways that lead to both medical mistrust and belief in conspiracy theories is important for diagnosing causes of under-vaccination as well as addressing the root causes of mistrust.

Finally, despite ongoing work on the causes and effects of medical mistrust, reviews of the literature have highlighted basic questions that remain unanswered [10]. For example, medical mistrust often covaries with socioeconomic status, which is used as a statistical control in many studies. We know little about what drives medical mistrust within and between SES gradients, races, and ethnic groups. In the US, most quantitative and qualitative work focuses exclusively on ethnic and gender minorities, and low socio-economic status groups. This makes it difficult to disentangle causal pathways between SES, race/ethnicity, stigma and discrimination, medical mistrust, and health outcomes. There is also little work comparing the most commonly used types of medical mistrust questionnaires, and a general lack of clarity on when and where these types of surveys should be applied. Finally, basic questions remain regarding the relationships between individual, group-based, and cultural drivers of mistrust, and how these differentially affect health behavior.

## Moving Forward

Addressing the vaccination gap in LMICs will require significant investment in understanding medical mistrust and its proximate determinants across diverse communities. More qualitative and quantitative data is needed on experiences with and beliefs about healthcare that can point us toward a better understanding of the drivers of medical mistrust. In particular, more research is needed on how norms, beliefs, and day-to-day experiences with healthcare systems can manifest in medical mistrust. Previous work [10] suggests that this is likely to include systemic factors (e.g. long-wait times, systemic discrimination), interpersonal factors (negative interactions with doctors and nurses), and vicarious factors (group-level historical suspicion and mistrust, intergenerationally transmitted information). Second, the development of a validated survey protocol that is applicable to a broad range of communities is sorely needed. New, validated instruments are necessary to capture the diversity of countries referenced here, including communities from the very rural to the very urban, communities that rely on traditional subsistence to those that are fully market integrated, communities with varying levels of education, communities that have suffered under past colonial rule or current levels of corruption and mismanagement, and across communities that may represent many ethnolinguistic groups. This is no small feat, but instruments in other areas of public health can serve as useful examples [42, 43].

In this “golden age of vaccines” addressing medical mistrust is a critical step toward reducing disease burdens and health disparities. The current focus on the “infodemic” only addresses a small piece of the problem because mistrust underlies information processing and healthcare decision-making. Addressing the underlying issues leading to mistrust is crucial. To do this we need better tools and a more nuanced understanding of what drives medical mistrust across diverse communities. Understanding the context of medical mistrust can inspire solutions, identify at-risk communities, and inform public messaging campaigns, helping us to make the most of new and rapidly emerging vaccine technologies.

## Data Availability

No datasets were generated or analysed during the current study.
